# Angiogenesis-related genes and immune microenvironment in moyamoya disease: a transcriptomic and functional analysis

**DOI:** 10.1186/s13023-025-03945-4

**Published:** 2025-07-28

**Authors:** Zhenyu Zhou, Hongchuan Niu, Shaoqi Xu, Junze Zhang, Yutong Liu, Chengxu Lei, Shihao He, Yuanli Zhao

**Affiliations:** 1 Department of Neurosurgery, Peking Union Medical College and Chinese Academy of Medical Sciences, Peking Union Medical College Hospital, Beijing, 100730 China; 2 Department of Neurosurgery, Peking University International Hospital, Beijing, China; 3 Department of Neurosurgery, Beijing Tiantan Hospital, Capital Medical University, Beijing, 100070 China; 4 Suzhou Vocational Health College, Suzhou, 215009 Jiangsu China; 5 Department of Pathology, Stanford University School of Medicine, Stanford, CA 94305 USA

**Keywords:** Moyamoya disease, Angiogenesis, Immune, Multi-omics

## Abstract

**Background:**

Moyamoya disease (MMD) is a chronic, progressive occlusive cerebrovascular disease. It causes recurrent cerebrovascular stroke due to vascular closure and proliferation. An unclear pathophysiological mechanism is the most significant obstacle in the diagnosis and treatment of MMD.

**Method:**

This study prospectively included 10 MMD and 3 HC (healthy controls) participants in the discovery cohort. GSE189993 and GSE157628 were downloaded from the Gene Expression Omnibus (GEO) as validation cohorts, which included 32 patients with MMD and 20 HC. Angiogenesis-related genes were downloaded from GENECARD. Hub genes were selected by differential analysis and weighted correlation network analysis. Functional enrichment, immune infiltration, and metabolic pathway analyses and drug prediction mapping (Connectivity Map [CMap]) were performed.

**Result:**

Through differential analysis identified, 198 differentially expressed genes (DEGs), including 85 upregulated genes and 113 downregulated genes. In total, 238 angiogenesis -related genes were identified using WGCNA. Four hub genes were identified: TBC1 domain family member 9B (TBC1D9B), Phosphatidylinositol transfer protein beta (PITPNB), The ANK repeat and PH domain-containing protein 3 (ARAP3), and Ubiquitin-conjugating enzyme E2 E1 (UBE2E1). Four potential drugs were selected: calyculin A, H-9, parbendazole, and velnacrine. The results of multiple immune infiltration analyses collectively depicted the immune microenvironment characteristics of MMD.

**Conclusion:**

This study is the first to explore the mechanism by which angiogenesis related genes are involved in intimal hyperplasia in Moyamoya disease. TBC1D9B and ARAP3 may promote the pathological development of moyamoya disease through immune response, metabolism.

**Supplementary Information:**

The online version contains supplementary material available at 10.1186/s13023-025-03945-4.

## Introduction

Moyamoya disease (MMD) is a rare cerebrovascular condition in which the internal carotid artery and its branches are progressively occluded [1]. In MMD, the most prevalent symptom is cerebral infarction resulting from occlusion of the distal end of the internal carotid artery [2].

Obstruction and proliferation of blood vessels lead to repeated occurrences of ischemic and hemorrhagic strokes. This leads to severe functional deficits in patients with MMD, such as cognitive dysfunction, hemiplegia, and language impairment [2]. Indirect cerebral revascularization using encephaloduroarteriosynangiosis creates new collateral vessels by forming vessels from the external to the internal carotid arteries in patients with MMD. Although this process has been extensively documented, there is a limited understanding of the mechanism behind the formation of these collateral vessels [3, 4]. Angiogenesis in MMD is crucial for pathogenesis and Indirect cerebral revascularization.

Angiogenesis is a complex physiological process involving various vascular cells and cytokines. Caveolin-1(Cav-1) promotes progressive vascular stenosis by regulating the angiogenic function of vascular endothelial cells [5]. The ring finger protein 213(RNF213) knockdown inhibited the overexpression of vascular endothelial growth factor receptor 2(VEGFR2) by affecting Yap/TAZ during pathological vascular proliferation [6]. In MMD, the physiological process of angiogenesis may occur through vessel occlusion, vascular proliferation, or collateral angiogenesis after indirect surgery. Although several studies have been conducted on angiogenesis-related genes in MMD, most are limited to the study of a single gene or protein. In a previous study, we analyzed the metabolic products (14 726 peptides and 1555 proteins) of 60 MMD patients and 20 healthy controls [7]. An imbalanced angiogenesis is the fundamental pathological process underlying the vascular occlusion and collateral vessel formation in moyamoya disease. RNF213, one of the most reliable moyamoya-related genes, has been reported to be closely related to angiogenesis in moyamoya disease [6]. However, mouse models based on RNF213 have not shown the formation of the characteristic moyamoya-like vasculature [8]. Caroline et al. suggest that moyamoya disease may be a complex immunovascular disease [9]. Therefore, a single factor’s pathogenesis mechanism may not apply to moyamoya disease. Angiogenesis is a multifaceted physiological process that involves the intricate interplay of numerous genes and cytokines. In addition, the interactions between genes and downstream pathways should be considered. However, few studies have investigated the effects of multiple angiogenesis-related genes on MMD.

In this study, we included ten patients with MMD and three healthy controls (HC) as the discovery cohort. Superficial temporal artery (STA) samples were collected from the discovery cohort and RNA sequencing was performed to obtain the gene dataset. GSE189993 and GSE157628 were downloaded from GEO as validation cohorts. Differential analysis was performed between the discovery and validation cohorts for the MMD differentially expressed genes (DEGs), including 85 upregulated and 113 downregulated genes. Angiogenesis-related genes were downloaded from the GENECARD. Using weighted correlation network analysis (WGCNA), we identified 238 angiogenesis-related genes with the strongest correlation with MMD. We extracted four genes from the 198 DEGs and 238 angiogenesis-related genes as hub genes for further study. Gene Ontology (GO) and Kyoto Encyclopedia of Genes and Genomes (KEGG) functional analysis, immune infiltration analysis and metabolic pathway analyses were performed on the hub genes. In conclusion, identifying angiogenesis-related genes may provide new insights into MMD pathogenesis.

## Method

### Participants and gene expression data

The discovery cohort included gene expression data from 13 patients, including three healthy controls and ten patients with MMD. This study was approved by the Institutional Ethics Committee of Peking Union Medical College Hospital, Beijing, China (I-24YSB0160). It is confirmed that all experiments were performed in accordance with relevant guidelines and regulations. All participants agreed to participate in the study and provided written informed consent. STA samples were obtained from patients during surgery. STA samples were preserved at − 80 °C for embedding in paraffin and stained with hematoxylin and eosin (HE). RNA sequencing was performed on STA samples to generate a gene expression database for patients. The GSE189993 gene expression database, which includes 32 patients (11HC, 21MMD), was downloaded from the NCBI GEO public database. The GSE157628 gene expression database, which included 20 patients (nine HC and 11 patients with MMD), was downloaded from the NCBI GEO public database. Both GSE189993 and GSE157628 were based on gene expression data from the middle cerebral artery (MCA) of patients with MMD (Supplementary Table S1).

### Differential expression analysis

To eliminate the batch effect among the three queues, SVA algorithm was used for correction. The correction results were displayed in a PCA chart. The batch effect was corrected using the ComBat function. A matrix containing only the intercept term was set up. The correction effect was verified through PCA. Differential analysis was conducted by fitting the expression data with a linear model. A contrast matrix was set up to compare MMD vs. HC. The eBayes shrinkage variance was applied to improve the robustness of small sample size for differential analysis. Limma analysis was used to detect DEGs between individuals with MMD and healthy controls in both the discovery and validation cohorts. The criteria for identifying DEGs were set as *P* value < 0.01 and |logFC|> 1.5. A volcano plot and heatmap were generated based on the results of the differential analysis to visualize the identified DEGs. The results were plotted using R software (version 4.2.2, https://www.r-project.org/), the SVA and Limma packages.

### Weighted gene co-expression network analysis

In order to identify the angiogenesis-related genes with the highest potential relevance in the pathogenesis of MMD, WGCNA was selected as the main analysis method. By employing the WGCNA-R algorithm, a gene expression network was established by analyzing weighted gene co-expression. The top 5,000 genes exhibiting the greatest variability were chosen, and a soft threshold of 5 was applied. The weighted adjacency matrix was transformed into a topological overlap matrix (TOM) to assess the network connectivity. Subsequently, hierarchical clustering is used to construct a tree structure based on the TOM matrix. Different branches within the clustering tree correspond to distinct gene modules, each represented by a different color. Genes were categorized according to their weighted correlation coefficients, which led to the grouping of genes with similar expression patterns into specific modules. Consequently, all the genes were segregated into multiple modules based on their respective expression patterns. The results were plotted using the WGCNA software package run by R software (version 4.2.2, https://www.r-project.org/).

### The functional annotation of GO and KEGG

In order to explore the connection between DEGs and MMD, it is necessary to explore the physiological functions of DEGs with higher weight. The Metascape database was used to functionally annotate the differentially expressed genes. Pathway analysis of specific genes was performed using the GO Min overlap ≥ 3 & *p* ≤ 0.01 is defined as statistically significant. The results are visualized on the Metascape web page (https://metascape.org/gp/index.html).

### Immune cell infiltration analysis

To explore the role of immune factors in MMD, immune infiltration analysis was used to depict the immune microenvironment in MMD. In this study, the single-sample Gene Set Enrichment Analysis (ssGSEA) algorithm was used to examine the immune cell types present in the microenvironments of patients with MMD. Initially, a quantitative analysis of immune cells in the gene expression database of patients with MMD was conducted, encompassing 29 human immune cell phenotypes, including T, B, and NK cells. Subsequently, based on the quantitative analysis results, estimations were made regarding the relative proportions of the 29 infiltrating immune cells were estimated. Pearson’s correlation analysis was performed to assess the relationship between gene expression and immune cell content. The results were plotted using the CIBERSORT algorithm and the ggplot2 software package run by R software (version 4.2.2, https://www.r-project.org/).

### Analysis of transcriptional regulation of hub genes

In this study, the “RcisTarget” was used to make predictions regarding transcription factors. Calculations performed using RcisTarget were based on motifs, and the normalized enrichment score (NES) of each motif was affected by the total number of motifs in the database. In addition to known motifs in the source data, annotation files were generated through inferences based on motif similarity and gene sequences. To assess the overexpression of each motif in the gene set, the area under curve (AUC) for each pair of motif-motif sets was calculated using a recovery curve approach. Analyze the sample of matching group information and key gene expression data. Fit a Logistic regression for each key gene. Calculate the predicted probability using predict (model_LR, type = “response”). Use pROC to calculate the AUC and 95% confidence interval, and draw the ROC curve. The NES for each motif was determined based on the AUC distribution across all motifs within the gene set. The “RcisTarget” was run by R software (version 4.2.2, https://www.r-project.org/).

### Connectivity map (CMap) drug prediction

In order to explore ways to correct the pathological angiogenesis that occurs during the progression of moyamoya disease, CMap was selected to screen potential drugs. CMap is a gene expression database developed by the Broad Institute that is based on the perturbation of gene expression and is primarily used to explore the role of small-molecule drugs in gene-disease relationships. It contains gene chip data from the expression profiles of over 1000 small molecule drugs applied to various human cell types. Experimental conditions such as temperature, time, and humidity can be simulated or determined. In this study, differentially expressed genes from the gene database of MMD were used as the basis for the predictive analysis of small-molecule drugs. Use PubChem Web to generate drug prediction results. (https://pubchem.ncbi.nlm.nih.gov/).

### Construction of overexpressed endothelial cell model

Specific primers were designed for the CDs region sequence of the target gene ARAP3. According to the multiple cloning site of the pLVX-IRES-Puro vector, EcoR I (GAATTC) and Xba I (TCTAGA) two restriction sites were added upstream and downstream of the primers respectively. The above sequence was amplified, purified and the PCR product with EcoR I and Xba I double restriction sites was recovered. ARAP3 primer sequences: Forward 5- GGAATTCATGGCTGCCCCTCAGGACCTGGACA -3′; Reverse 5′- GCTCTAGACTCATGTGAGGGGCTGGCTGGAG -3′. After PCR identification, plasmid extraction, lentivirus packaging, lentivirus titer detection and lentivirus transfection, the transfection efficiency was verified by PCR experiment.

### ELISA detection of ARAP3 in serum

Prepare the standard solution according to the reagent instructions. The concentrations of TNF-α standard solutions are 10, 5, 2.5, 1.25, 0.625, 0.312, and 0.156 ng/mL in sequence. Take the microplate strips and put the unused strips back into the aluminum foil bag and seal it, then store it at 4 °C. Set up standard wells and sample wells on the pre-coated plate, and add 100 μL of standard solution or sample to each well. Seal the plate and incubate at 37 °C for 60 min. Discard the liquid and add 100 μL of detection reagent A to each well, seal the plate, and incubate at 37 °C for 1 h. Discard the liquid in each well and add 350 μL of washing solution to each well, and wash the plate three times. Add 100 μL of detection reagent B to each well, seal the plate, and incubate at 37 °C for 90 min. Discard the liquid in each well and add 350 μL of washing solution to each well, and wash the plate five times. Add 90 μL of TMB activator to each well and incubate at 37 °C for 10 min in the dark. Add 50 μL of stop solution to each well and mix well. Use an enzyme-linked immunosorbent assay (ELISA) reader set at 450 nm to measure the optical density value of each well within 30 min.

### Tube formation

The day before the experiment, take the Matrigel gel from the – 20 °C refrigerator and place it in a 4 °C refrigerator to thaw overnight. The day before the experiment, place the pipette tips in a −20 °C refrigerator to pre-cool them. Take them out 30 min before the experiment and place them on ice. Add 50 μL of the thawed Matrigel gel to each well of a 96-well plate. Do not generate bubbles during the addition process. Gently shake the plate to spread the gel evenly and incubate at 37 °C in an incubator for 30 min to allow the Matrigel gel to fully solidify. Take HBMEC cells with a fusion degree of 70–80% and in the logarithmic growth phase, digest them with trypsin, centrifuge for 3 min, resuspend them in complete medium, and count them. Add the cells at a density of 1.5 × 104 cells/well to the 96-well plate. Do not touch the gel surface and gently mix the cell suspension to avoid excessive differences in cell distribution within the group. Observe under a microscope to ensure the cells are evenly distributed. Gently shake the 96-well plate until the cells are evenly spread. Set up three replicate wells for each well and place them in a 37 °C incubator for routine culture for 6 h. Closely observe the formation of blood vessels after 2 h of culture. After 6 h of culture, when the lumen is completely formed, take out the 96-well plate and take pictures under a 100 × microscope. Use Image J software to analyze the total length of the tubes. Repeat the experiment three times.

### Statistical analysis

All statistical analyses were conducted using the R software (version 4.2.2, https://www.r-project.org/). All statistical tests were two-sided, and *p* < 0.05 was considered statistically significant.

## Result

### The acquisition of gene expression data

**Fig. 1:**
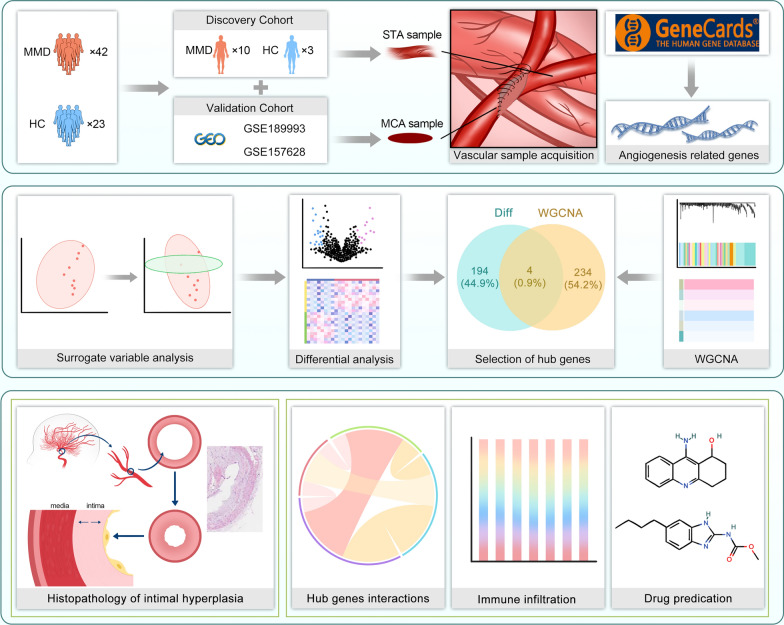
Research framework of this study. Differential analysis was performed on the gene datasets of the discovery and validation cohort to identify differentially expressed genes. Angiogenesis-related genes obtained from GeneCard were subjected to WGCNA. The intersection of the differentially expressed genes and angiogenesis-related genes was extracted to obtain hub genes. Subsequently, interaction analysis, immune infiltration analysis and drug prediction were conducted based on the hub genes. In in vitro experiments, intimal hyperplasia and abnormal internal elastic membrane structure were observed in the STA of patients with MMD

**Fig. 2:**
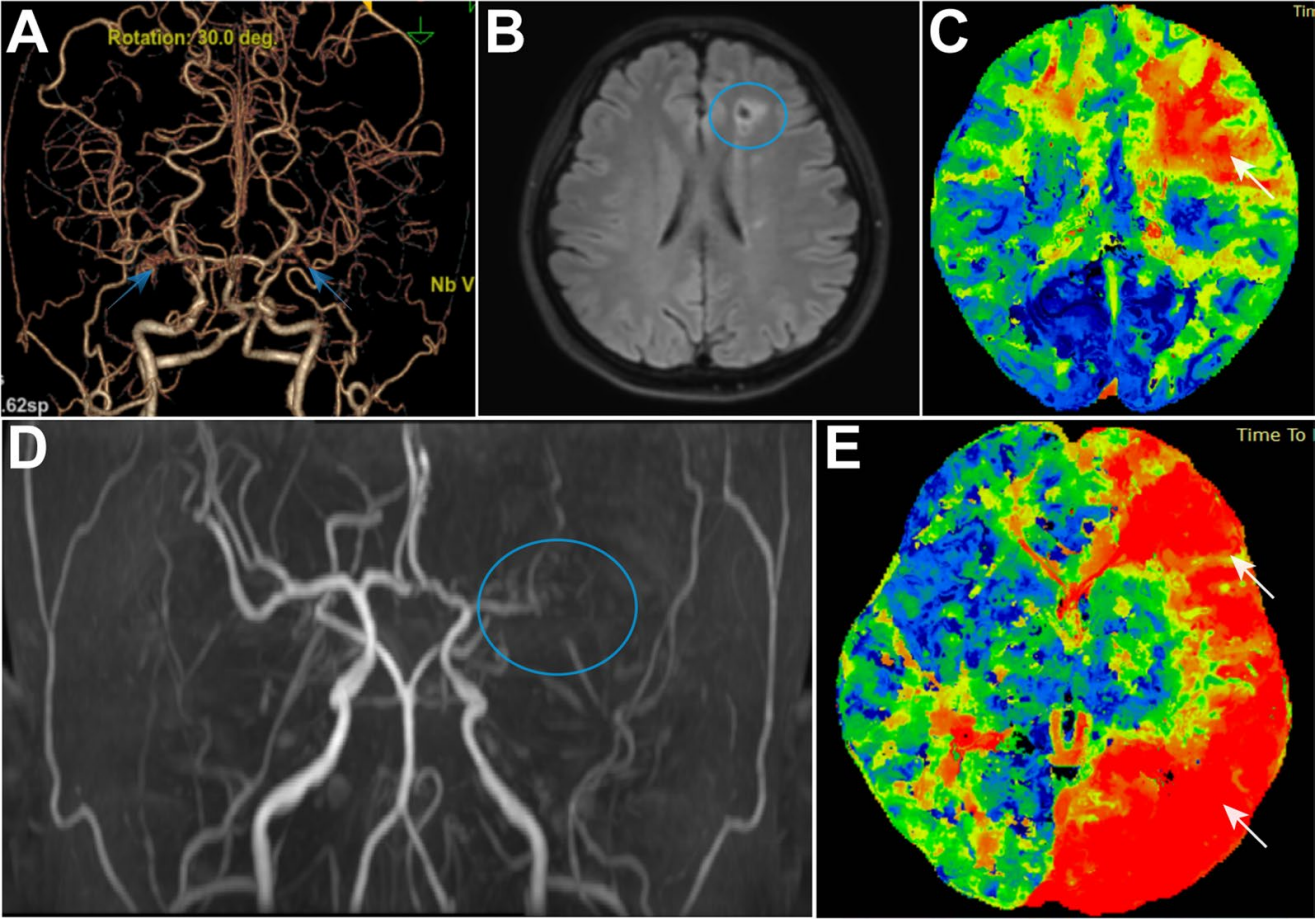
Typical image of bilateral and unilateral Moyamoya disease. **A** MRA images of cerebral vessels in a patient with bilateral Moya Moya disease. The blue arrow indicates the occluded middle cerebral artery. **B** MRI images of cerebral infarction in a patient with Moya Moya disease. The blue circle indicates the area of cerebral infarction. **C** Cerebral perfusion images of a patient with Moya Moya disease. The red area represents the low perfusion region, which coincides with the location of cerebral infarction. **D** CTA images of cerebral vessels in a patient with unilateral Moya Moya disease. The blue circle indicates the occluded middle cerebral artery. **E** Cerebral perfusion images of a patient with unilateral Moya Moya disease. The red area represents the low perfusion region, which is consistent with the side of the occluded middle cerebral artery

In the discovery group, 10MMD and3 HC were included. The STA samples were obtained from patients undergoing bypass surgery. Using RNA sequencing, gene expression data were obtained from 13 patients (ten patients with MMD and three HC). In the validation cohort, gene expression data for MMD (GSE189993 and GSE 157628) were downloaded from the GEO database, including the gene expression data for the MCA of 55 patients (32 patients with MMD and 20 HC). A total of 42 MMD and 23 HC gene expression datasets were analyzed (Fig. 1). The clinical information of the participants is presented in table form and provided in the supplement (Tables S1, S2). The characteristic imaging results of MMD are shown in Fig. 2.

### The 198 differential expression genes of moyamoya disease

**Fig. 3:**
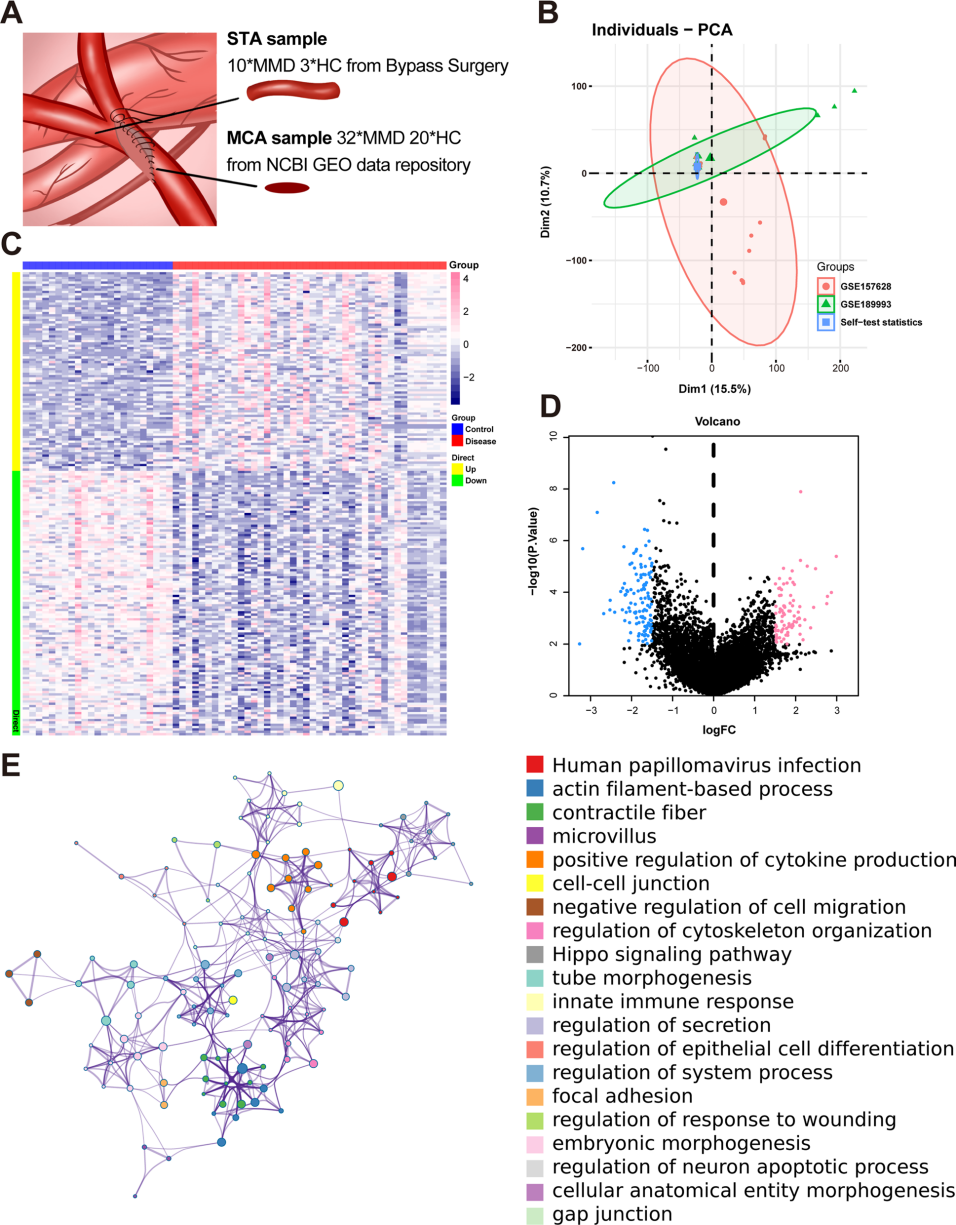
Differential analysis of the gene database for MMD. **A** STA and MCA samples were obtained during bypass surgery. STA: superficial temporal artery. MCA, middle cerebral artery; MMD, moyamoya disease; HC, healthy control. **B** PCA plots of the validation and discovery groups after SVA correction. Red: GSE157628, green: GSE189993, blue: discovery group. **C** Heatmap of differentially expressed genes in the MMD gene database. Red: group, blue: control group. **D** Volcano plot of DEGs in the MMD gene database. Red: upregulated genes, blue: downregulated genes. **E** Network diagram of functional enrichment analysis of DEGs

**Fig. 4:**
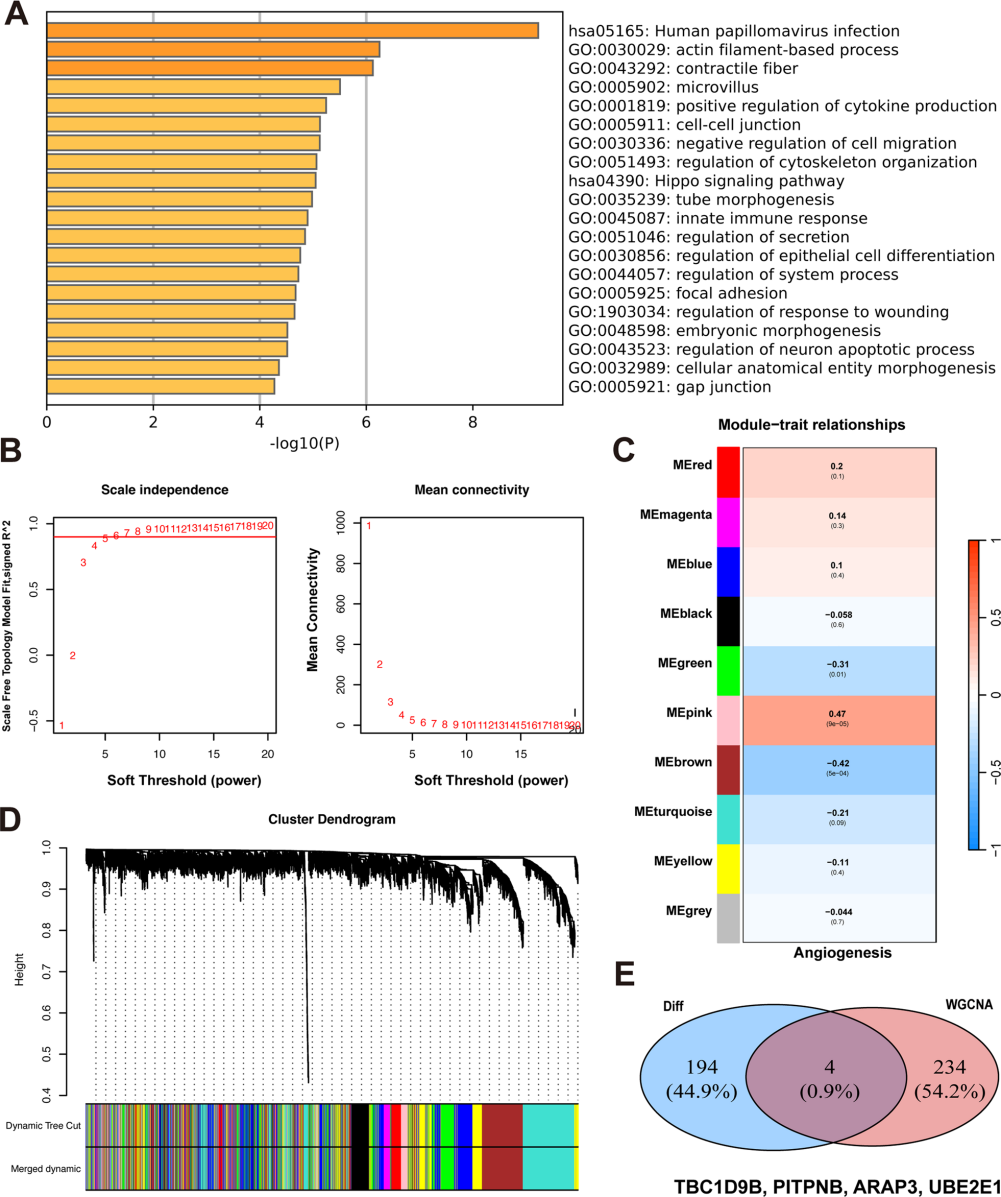
Weighted gene co-expression network analysis of angiogenesis-related genes. **A** Bar chart of functional enrichment analysis of DEGs. **B** Scale-free fitting index (left) and average connectivity (right) for different soft-thresholding powers β. The red line represents the correlation coefficient of 0.9. **C** Different blocks represent different gene modules (left panel). Different blocks represent the correlations between gene modules and diseases (right panel). **D** Hierarchical clustering dendrogram of co-expression modules, with different colors representing different modules. **E** Venn diagram showing overlap between DEGs (blue) and angiogenesis-related genes (red)

After applying the SVA algorithm for correction, the batch effects in the gene expression databases decreased (Fig. 3B). The DEGs were filtered based on a *P* value < 0.01 and |logFC|> 1.5 criteria, resulting in 198 identified DEGs, comprising 85 upregulated genes and 113 downregulated genes (Fig. 3C, D). The expression of 198 DEGs is shown in the supplementary material. (Table S3) Metascape analysis indicated that the differentially expressed genes were mainly linked to the enhancement of cytokine production, the Hippo signaling pathway, and pathways related to cell–cell junctions (Figs. 3E, 4A).

### The identifying four angiogenesis-related hub genes

Download angiogenesis-related genes from the GenBank database. The levels of angiogenesis-related genes were quantified using ssGSEA, and a WGCNA network was constructed (Fig. 4B). The soft threshold β was established at five gene modules for the detection of gene modules using the TOM matrix. In this analysis, ten gene modules were identified: black (273), blue (940), brown (834), green (530), gray (18), magenta (119), pink (238), red (284), turquoise (1025), and yellow (739) (Fig. 4C, D). Disease showed the strongest correlation with the pink module (cor = 0.47, *p* = 9e-05). By identifying the intersection of genes related to the pink module and DEGs, four hub genes were identified: *TBC1D9B*, *PITPNB*, *ARAP3*, and *UBE2E1* (Fig. 4E).

### Interactions and diagnostic prediction of hub genes

**Fig. 5:**
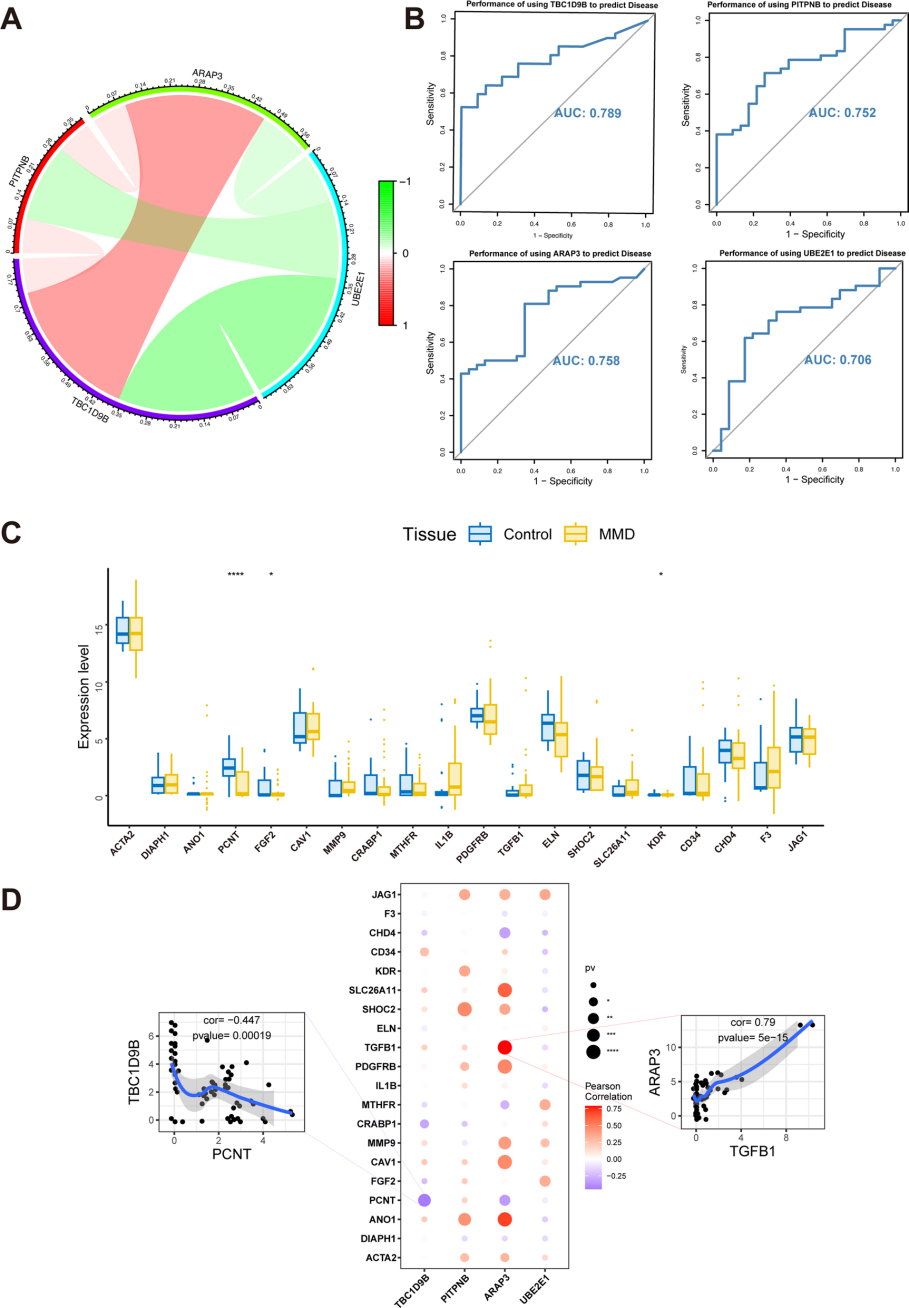
Correlation analysis diagnostic prediction efficacy of hub genes. **A** Correlation analysis of the hub genes. Green: ARAP3, blue: UBE2E1, purple: TBC1D9B, red: PITPNB. **B** Diagnostic prediction efficacy analysis of hub genes. **C** Comparison of moyamoya-related genes between control and disease groups. Blue: control group, yellow: disease group. **D** Correlation analysis of the hub and moyamoya-related genes. Horizontal axis: hub genes, vertical axis: moyamoya-related genes

Based on the gene expression data, a network of mutual interactions was constructed among the hub genes (Fig. 5A). The diagnostic ability of hub genes was evaluated using ROC curves. The results show the AUC values of the hub genes. *ARAP3* AUC = 0.758, *PITPNB* AUC = 0.752, *TBC1D9B* AUC: 0.789, and *UBE2E1* AUC: 0.706 (Fig. 5B). MMD-associated genes were obtained from the GeneCards database, and their expression variances were examined. The expression levels of *PCNT*, *FGF2*, and *KDR* were significantly different between the control and disease cohorts (Fig. 5C). The expression levels of the hub genes were significantly correlated with the expression levels of numerous genes related to MMD (Fig. 5D). *ARAP3* expression was significantly and positively correlated with *TGFB1* expression (Pearson’s r = 0.79). *TBC1D9B* was significantly and negatively correlated with *PCNT* levels (Pearson’s r = 0.447).

### The superficial temporal artery intimal thickening cause hemadostenosis

**Fig. 6:**
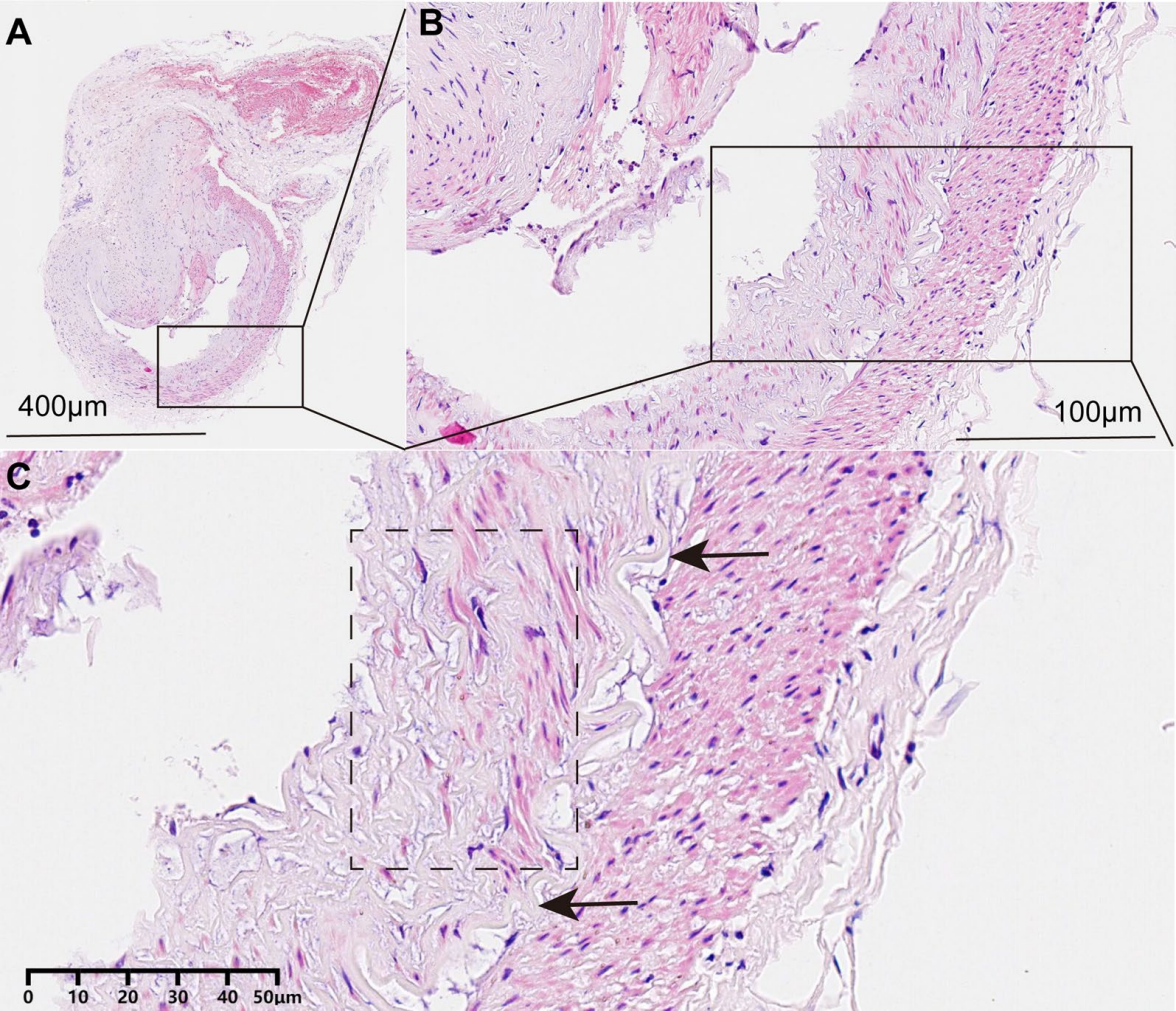
The superficial temporal artery intimal thickening cause hemadostenosis. **A** In moyamoya disease, the lumen of the vessel was markedly narrowed. Scale bar: 400 µm. **B** The vessel wall of the stenotic vessel was significantly thickened. Scale bar: 100 µm. **C** The intimal is hyperplasia and cells were disorderly arranged. The structure of the internal elastic membrane was changed. Scale bar: 50 µm

In patients with MMD, the lumen of the STA is significantly narrowed and the wall of the STA is thickened (Fig. 6A). In the thickened wall of STA, the intimal hyperplasia is particularly prominent in which may be one of the main causes of stenosis (Fig. 6B). Under magnified view, the cells in the intimal hyperplasia were disorderly arranged, and the structure of the internal elastic membrane was changed (Fig. 6C).

### Characteristics of immune microenvironment in moyamoya disease

**Fig. 7:**
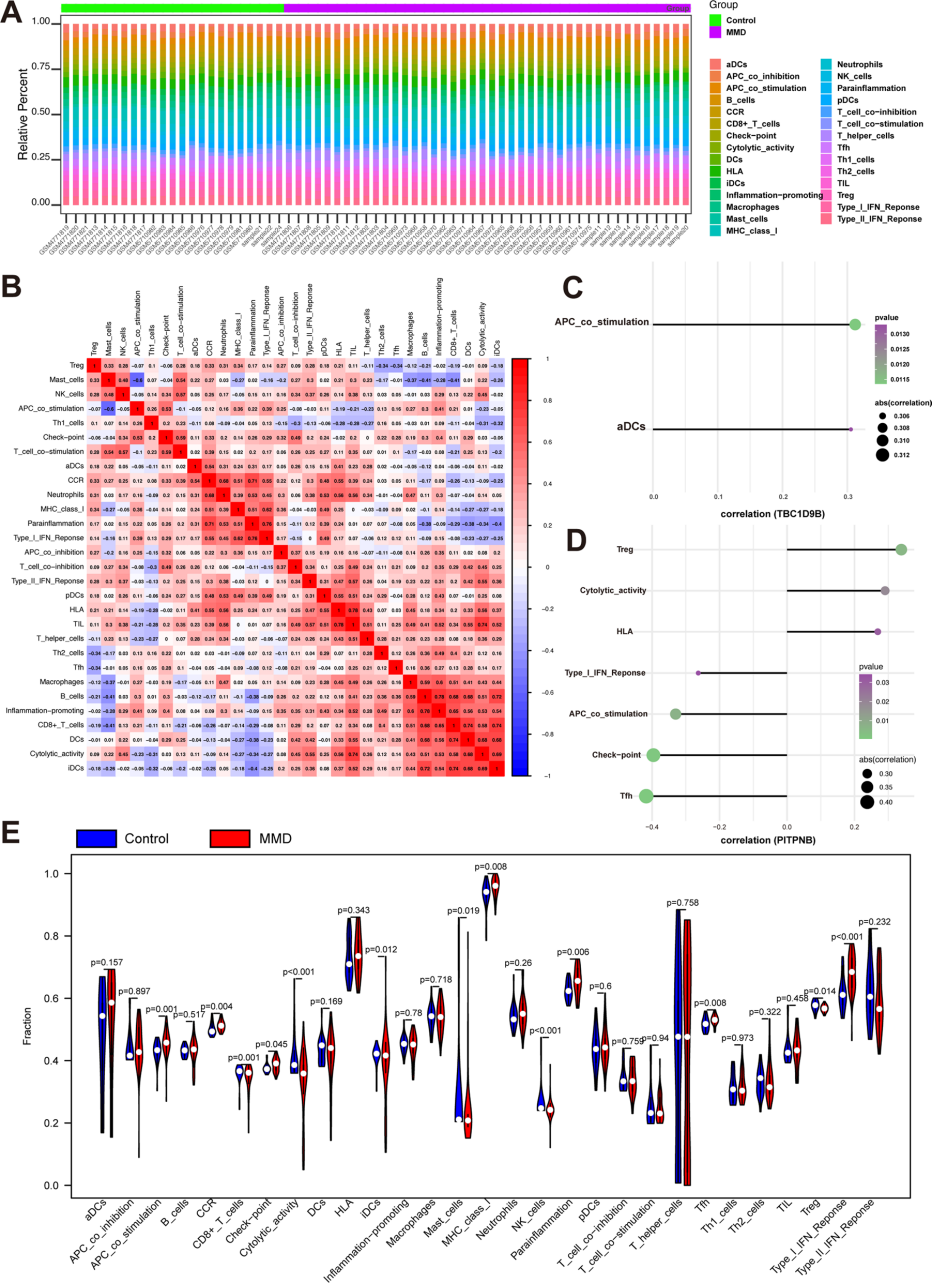
Immune infiltration analysis. **A** Analysis of immune cell proportions. Green: control group, purple: disease group, horizontal axis: number of patients, vertical axis: proportion of immune cells. **B** Correlation analysis of immune cells. **C** Correlation analysis of TBC1D9B with immune cells. **D** Correlation analysis of PIRPNB with immune cells. **E** Comparison of immune cells between control and disease groups. Horizontal axis: name of immune cells, vertical axis: standardized score of immune cell expression levels, blue: control group, red: disease group

Through an examination of the connections between hub genes and immune infiltration, this investigation delves into the potential molecular pathways through which pivotal genes impact the progression of MMD. This illustrates the distribution of immune infiltration levels and demonstrates the interrelationships among immune cells (Fig. 7A, B). The interaction between hub genes and immune cells were analyzed: TBC1D9B significantly positively correlated with APC_co-stimulation and dendritic cells (aDCs) (Fig. 7C), and PITPNB is significantly positively correlated with regulatory T cells (Tregs), Cytolytic_activity, and HLA, and negatively correlated with Tfh, check-point, and APC_co-stimulation (Fig. 7D). In addition, ARAP3 isignificantly positively correlated with Tfh, B_cells, and aDCs, and negatively correlated with Tregs (Fig. S1A). UBE2E1 significantly negatively correlated with MHC_class__I, pDCs, and CCR (Fig. S1B). Furthermore, the APC_co_stimulation, CCR, and checkpoints differed significantly between the two groups (Fig. 7E). The gene set used in this analysis comprised hub genes regulated by multiple mechanisms involving transcription factors. Enrichment analysis was performed using the cumulative recovery curves. Examination of significant genes and motif-TF annotation revealed that the NES was highest (NES: 8.85) for the cisbp M0326 motif. A network diagram depicting the motifs of the hub genes and their corresponding transcription factors was generated (Fig. S2).

### Metabolic pathway relatedness to hub genes

**Fig. 8:**
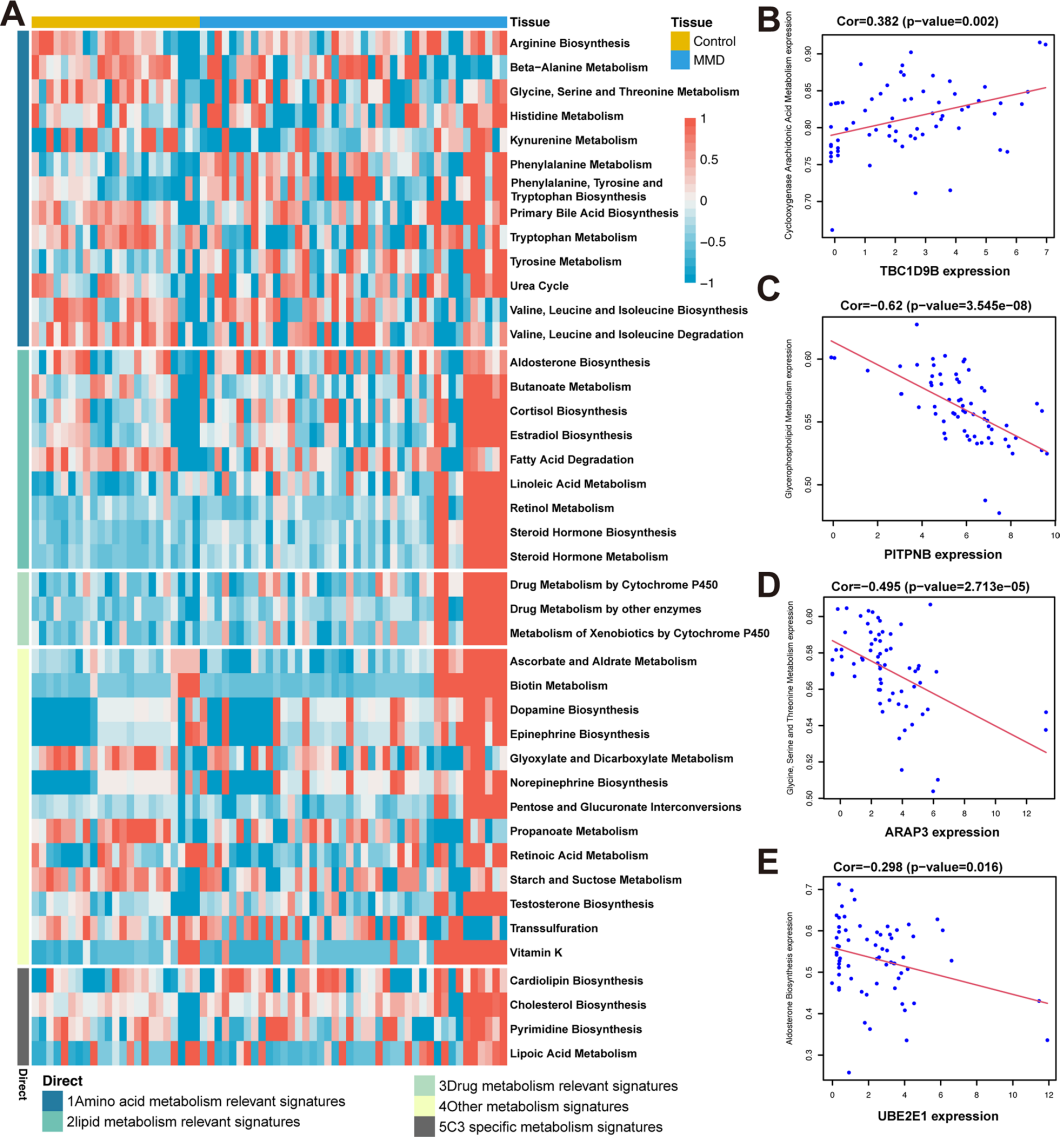
Metabolic pathway analysis of hub genes. **A** Metabolic pathway analysis of control group vs. disease group. **B** Correlation analysis between TBC1D9B and cyclooxygenase arachidonic acid metabolism expression. **C** Correlation analysis between PITPNB and glycerophospholipid metabolism expression. **D** Correlation analysis between ARAP3 and glycine, and serine and threonine metabolism expression. **E** Correlation analysis between UBE2E1 and aldosterone biosynthesis expression

To explore the connections between hub genes and metabolic pathways, metabolic pathway scores were quantified using ssGSEA. The association between the hub genes and metabolic pathways was visualized using a heatmap and scatter plot (Fig. 8A–E).

### Four potential drugs of moyamoya disease

The top 50 genes that are upregulated and downregulated into two categories. A Connectivity Map database was used for drug prediction. The results showed that the expression profiles disrupted by drugs such as calyculin A, H-9, parbendazole, and velnacrine negatively correlated with the expression profiles disrupted by the disease. This implies that these medications have the potential to alleviate the disease.

### The effect of ARAP3 on endothelial cell tube formation function

**Fig. 9:**
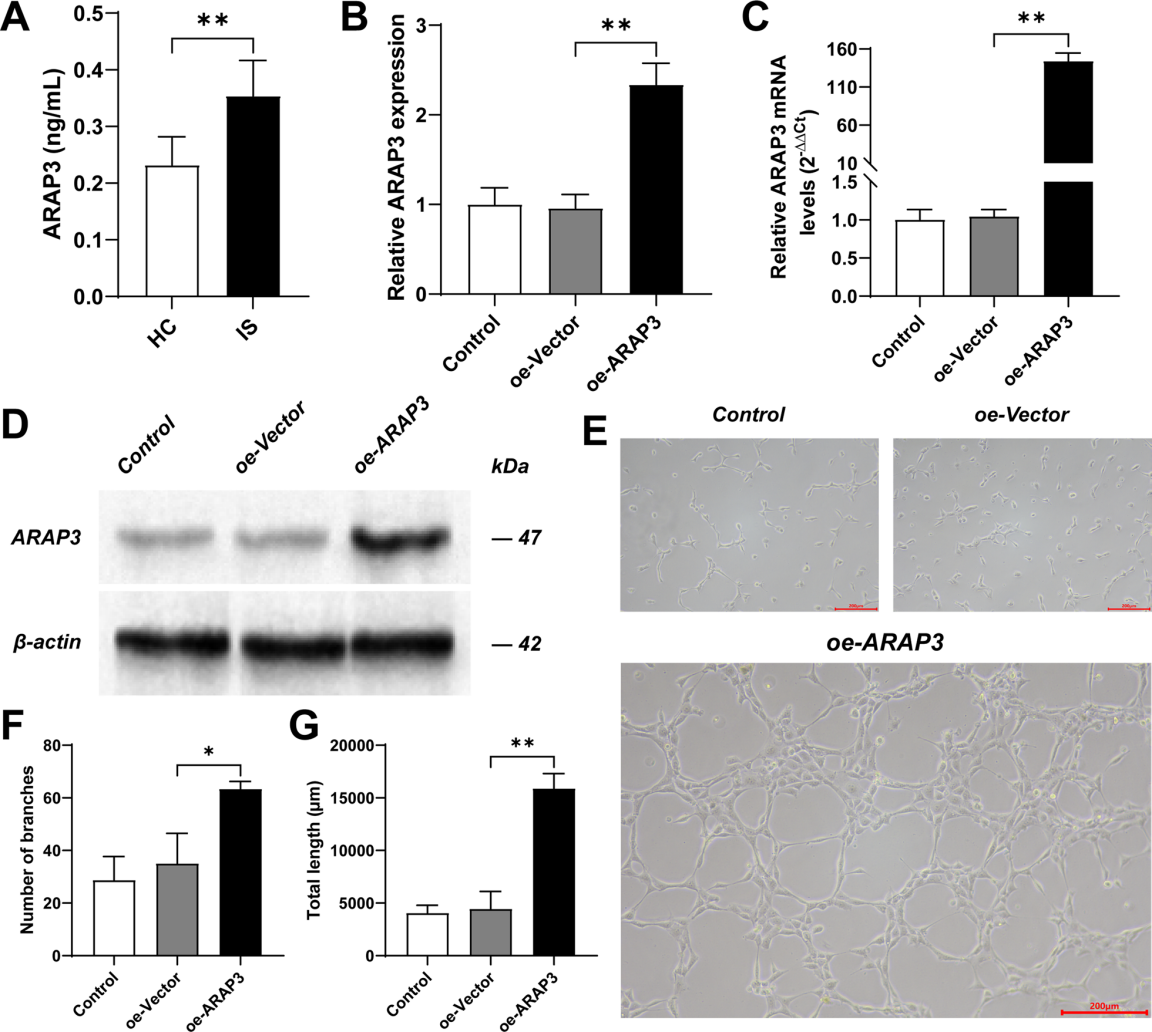
The expression level of ARAP3 and its effect on the formation of endothelial cell tubes. **A** The contents of ARAP3 (A) in the serum were measured by ELISA assay. Results were mean ± SD for five individual experiments. *p < 0.05, **p < 0.01. **B** Detection of ARAP3 expression efficiency in endothelial cells. The expression level of ARAP3 in the oe-ARAP3 group was significantly higher than that in the control group and the oe-vector group. *p < 0.05, **p < 0.01. **C** Detection of ARAP3 mRNA expression efficiency in endothelial cells. The expression level of ARAP3 mRNA in the oe-ARAP3 group was significantly higher than that in the control group and the oe-vector group. *p < 0.05, **p < 0.01. **D** Western Blot of ARAP3 expression efficiency in endothelial cells. **E** The angiogenesis potential of ECs in each group was evaluated through a tubule formation assay. Bar = 200 μm. **F** Bar chart of the number of tubules. The number of tubules in the oe-ARAP3 group was significantly higher than that in the oe-vector group and the control group. *p < 0.05, **p < 0.01. **G** Bar chart of the length of tubules. The length of tubules in the oe-ARAP3 group was significantly higher than that in the oe-vector group and the control group. *p < 0.05, **p < 0.01

The ELISA results showed that the expression level of ARAP3 in the serum of patients with cerebral aneurysm disease was significantly higher than that of the control group (Fig. 9A). The expression level of ARAP3 in the endothelial cell model constructed by lentivirus transfection was significantly higher than that of the blank group and the control group (Fig. 9B–D). In the tubule formation experiment, the number and length of tubules in the endothelial cells overexpressing ARAP3 were significantly higher than those in the blank group and the control group (Fig. 9E–G).

## Discussion

MMD is a vascular occlusion disease with an unknown pathogenesis that hinders diagnosis and treatment. In this study, the four most promising angiogenesis-related genes were selected as hub genes in MMD using differential analysis and WGCNA. To elucidate the roles of hub genes in MMD, immunohistochemistry, GO and KEGG [10–12] functional enrichment, and metabolic pathway analyses were conducted.

In previous studies of the STA in patients with MMD, this research team identified the pathological expression of basic fibroblast growth factor (bFGF) [13]. This investigation elucidates a novel molecular mechanism underlying intimal thickening in MMD through analysis of STA specimens. Shoujia et al. assessed the intimal thickness of both the MCA and STA in 30 MMD patients and 12 control subjects. The findings indicated that MMD patients exhibited significantly thicker intima in the STA, mirroring observations made in the MCA [14]. Typical pathological features associated with moyamoya disease, such as endometrial thickening, were also evident in our hematoxylin–eosin (HE) staining sections of the STA.

The ANK repeat and PH domain-containing protein 3 (*ARAP3*) is a GTPase-activating protein that primarily regulates RhoA and Arf [15, 16]. In angiogenesis involving endothelial cells, the regulation of *ARAP3* signaling is essential for angiogenesis in endothelial cells. Vascular defects in angiogenesis have also been observed in *ARAP3* knockout mice [15, 17]. The active angiogenesis of MMD confirms the abnormal upregulation of *ARAP3* in the results of differential analysis. Therefore, abnormal upregulation of *ARAP3* during angiogenesis may be involved in the pathogenesis of MMD. Transforming growth factor beta-1 (TGFB1) regulates angiogenesis and is closely associated with MMD in Europe [18]. In this study, TGFB1 was upregulated in the disease group compared to the control group. Moreover, *ARAP3* expression positively correlated with TGFB1 expression. This suggests that *ARAP3* may promote the pathogenesis connected with TGFB1 in MMD.

TBC1 domain family member 9B (*TBC1D9B*) is a GTPase-activating protein that belongs to the Rab family [19]. In this study, *TBC1D9B* negatively correlated with pericentrin (*PCNT*). In a cohort study of familial intracranial aneurysms, *PCNT* was found that it may be a highly credible candidate gene for cerebrovascular diseases. Furthermore, different degrees of intracranial hemorrhage were observed in *PCTN* knockdown mice [20]. In MMD, the inhibition of *PCTN* by *TBC1D9B* may lead to similar pathological processes that cause cerebral infarction.

Phosphatidylinositol transfer protein beta (*PITPNB*) is a phospholipid that plays essential roles in the dynamics of cellular membranes and organization of the cytoskeleton, contributing to cellular signaling [21, 22]. In metabolic pathway analysis, it shows a negative correlation with glycerophospholipid metabolism. RNF213 and GUCY1A3 are regarded as potential pathogenic genes for Moyamoya disease. Meanwhile, they are also important lipid metabolism regulators, especially in terms of lipotoxicity, NF-κB-mediated inflammation and NO-mediated vascular protection [23]. In our previous study, we conducted non-targeted metabolic analysis on the serum of patients with Moyamoya disease. This is one of the most detailed studies on MMD metabolism, but the dataset did not focus on angiogenesis. Combined with in vitro experiments, we found that LPC can inhibit the abnormal cell viability and proliferation of HBVSMCs and the angiogenic function of HBMECs [24]. PITPNB may precisely play a synergistic role with other potential pathogenic genes of Moyamoya disease in lipid metabolism, jointly regulating the metabolism in Moyamoya disease. This study provides a new perspective on the mechanism underlying MMD in abnormal lipid metabolism.

Ubiquitin-conjugating enzyme E2 E1 (*UBE2E1*) belongs to the class of E3 ubiquitin ligases [25]. Ubiquitination is an important physiological process that maintains cellular homeostasis and regulates angiogenesis in various diseases. In liver cancer, *UBE2E1* serves as a marker of T cells [26]. Therefore, in MMD, *UBE2E1* may be involved in the T cell immune response and angiogenesis.

In vascular diseases, immune-mediated inflammation of the vasculature is a common cause of vascular narrowing and occlusion without atherosclerosis [27, 28]. MMD is also occlusive vascular disease without atherosclerosis. Moreover, in MMD, immune-related factors may play a role in altering vascular endothelial function and morphology, ultimately leading to endothelial thickening [29]. Describing the immune features in MMD helps differentiate MMD from vascular inflammation and assists in exploring the pathogenesis of MMD. Immune infiltration analysis showed significant differences (*p* < 0.05) in the expression of 13 immune cell types between the control and disease groups. The proportion of T cell phenotypes related to T cells was the highest. This suggests that an immune response dominated by T cells may exist in MMD. In the study on lysosome-related genes in moyamoya disease, they indicated that lysosome-related genes could serve as potential biomarkers for moyamoya disease. They found that the proportion of activated CD4 memory T cells, follicular helper T cells, and neutrophils was significantly increased in MMD. Moreover, the study reported that lysosome-related genes may directly participate in the pathogenesis of moyamoya disease and suggested that it may be indirectly promoted by regulating the immune system [30].In our previous study, we also found changes in immune factors: for example, the immune infiltration analysis showed that the HLA (*p* = 0.001), MHC I (*p* = 0.013), and type I IFN response (*p* < 0.001) were significantly higher in the MMD group than in the control group [31]. This trend is consistent with the immune factor changes found in this study for moyamoya disease. In our study, compared to the control group, Tfh was upregulated and Tregs were downregulated in the disease group. This is consistent with the results of immune cell correlation analysis of *ARAP3* and *PITPNB*. Additionally, *ARAP3*, *UBE2E1*, and *TBC1D9B* may influence immune cells, which could contribute to the upregulation of CCR and aDCs in MMD. In the study by Weng et al., 26 cases of MMD, 21 cases of atherosclerotic thrombotic stroke, and 32 healthy controls were included to analyze the association between the Treg/Th17 ratio and MMD [32]. They found that the Treg level in MMD was related to the MMD stage and might promote the onset of MMD by inducing VEGF. This suggests that Treg may contribute to the alteration of the immune microenvironment in MMD and thus be involved in the pathogenesis. In Jin et al.’s study, machine learning was employed to explore immune-infiltrated hub genes in Moyamoya disease as potential biomarkers [33]. They found that Treg was significantly decreased in Moyamoya disease compared with the control group in the immune infiltration analysis results. This phenomenon of decreased Treg expression is similar to the immune infiltration analysis results in this study. Additionally, hub gene ARAP3 showed a negative correlation with Treg. This suggests that overexpression of ARAP3 may be one of the reasons for the reduction of Treg in Moyamoya disease. Therefore, ARAP3 may not only be involved in the pathogenesis of Moyamoya disease by regulating the tube-forming function of endothelial cells but also by influencing the immune microenvironment. In MMD, *RNF213* mutations promote T-cell immune responses by enhancing antigen presentation [34]. This suggests that T cells can directly drive the onset of MMD through the immune response and indirectly contribute to the pathogenesis of MMD by affecting angiogenesis-related genes.

The synergistic effects of hub genes are evident in the immune system, as well as in functional and metabolic pathways. In the functionally enriched pathways of the DEGs, actin filament-based processes, regulation of cytoskeleton organization, and tube morphogenesis were highly enriched. Abnormalities in actin and the cytoskeleton have been suggested to promote endothelial thickening in MMD [35].

Four compounds were identified in the CMap analysis: calyculin A, H-9, parbendazole, and celnacrine. Among these, calyculin A relax vascular smooth muscles by inhibiting myosin light-chain phosphatase. This has a synergistic effect with the downstream signaling pathways of *ARAP3* and RhoA/Rho [36, 37]. Therefore, calyculin A may impede the progression of MMD by indirectly affecting the interaction between RhoA/Rho and vascular smooth muscle cells.

In conclusion, hub genes may participate in the pathogenesis of MMD by influencing immunity, metabolism, and angiogenesis. The promising hub gene *ARAP3* may be a key regulatory factor in the angiogenesis of MMD, and may also promote the immune response of T cells. Immune infiltration analysis showed that a T cell-dominated immune response in MMD may promote narrowing and occlusion of the vasculature. In the metabolic pathway, highly enriched cytoskeleton and actin levels reflect the potential mechanism of vascular endothelial narrowing in MMD. Angiogenesis-related genes may provide new insights into the study of MMD.

### Limitations of the study

The limited number of patients in the discovery cohort made it difficult to interpret the experimental results precisely. Although we included external data as a validation cohort, in future studies, it is still necessary to further expand the scale of the discovery cohort. Additionally, the control group of patients in the GEO database showed differences in disease. Animal models for validating the functions of hub genes are currently lacking. It is also difficult to carry out the detection tests for the safety and effectiveness of small molecule drugs. In addition, the lack of longitudinal data makes it difficult to explore the expression levels of hub genes over time. In future studies, we will expand the cohort size and apply more detailed longitudinal data to explore the role of angiogenesis-related genes in MMD.

## Conclusion

In this study, we downloaded public MMD data from the Gene Expression Omnibus (GEO) database as a validation cohort based on the discovery cohort. Differential analysis identified 85 upregulated and 113 downregulated genes. In total, 238 angiogenesis-related genes were identified using WGCNA. Four hub genes were identified: TBC1D9B, PITPNB, ARAP3 and UBE2E1. We characterized the immune microenvironment of patients with MMD using immune infiltration analysis. Furthermore, we explored the potential pathogenic mechanisms of MMD by functional and metabolic analyses based on hub genes. Angiogenesis-related genes represented by hub genes provide new insights into the mechanisms of MMD.

## Supplementary Information

Additional file1

## Data Availability

The databases (GSE189993 and GSE157628) are published and available in the GENE EXPRESSION OMNIBUS (https://www.ncbi.nlm.nih.gov/geo/info/datasets.html) [38]. The datasets generated and analysed during the current study are available in the GENE EXPRESSION OMNIBUS, [GSE189993, GSE157628]”.Additionally, any information presented in the current study are available from the corresponding author on reasonable request.

## Abbreviations

MMD: Moyamoya disease
GEO: Gene expression omnibus
CMap: Connectivity Map
DEGs: Differentially expressed genes
HC: Healthy controls
STA: Superficial temporal artery
GO: Gene ontology
KEGG: Kyoto encyclopedia of genes and genomes
HE: Hematoxylin and eosin
MCA: Middle cerebral artery
TOM: Topological overlap matrix
ssGSEA: Single-sample gene set enrichment analysis
NES: Normalized enrichment score
AUC: Area under curve
aDCs: Dendritic cells
Tregs: Regulatory T cells
ARA3: The ANK repeat and PH domain-containing protein 3
TBC1D9B: TBC1 domain family member 9B
PITPNB: Phosphatidylinositol transfer protein beta
UBE2E1: Ubiquitin-conjugating enzyme E2 E1
