# Supplementary material for: Angiogenesis-related genes and immune microenvironment in moyamoya disease: a transcriptomic and functional analysis
Source: Orphanet J Rare Dis. 2025 Jul 28;20:385. doi: 10.1186/s13023-025-03945-4 (PMC12306060; doi:10.1186/s13023-025-03945-4)
Supplement: Supplementary file 1 — Additional file1 [file 13023_2025_3945_MOESM1_ESM.pdf]

Figure S1

A: Immunocytochemical analysis of ARAP3

B: Immunocytochemical analysis of UBE2E1

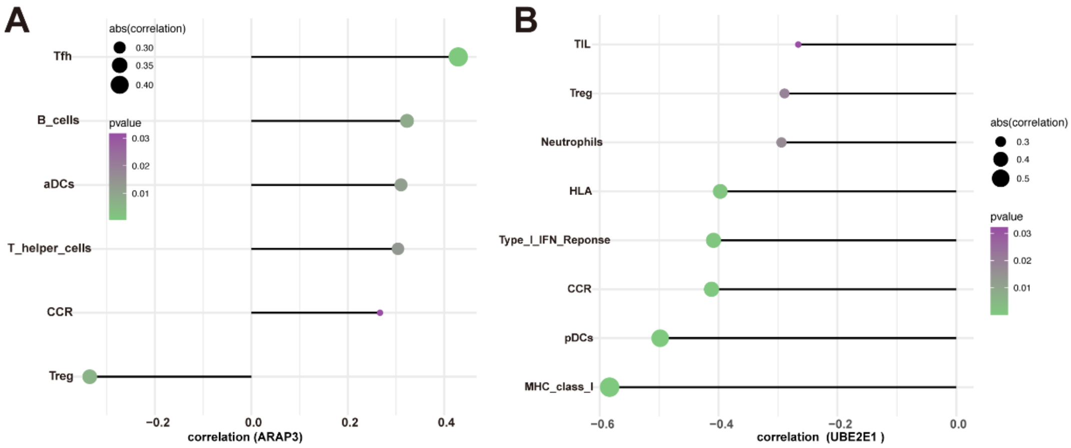

Figure S2: Regulatory network analysis of hub genes

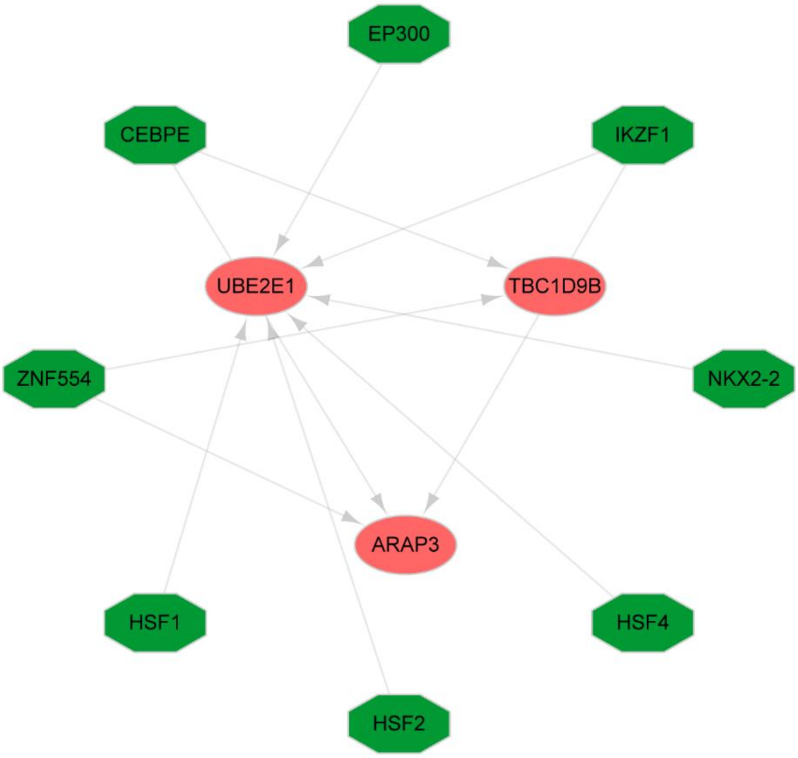

Table S1: Detailed clinical information for the participants of GSE189993 and GSE157628.

ID	disease	Age(year)	sex	Taken medicines	Antibiotics for the operation	Clinical presentation	Duration until surgery from the last clinical presentation	Subtype of MMD	Aneurysm location/size	Epileptic origin
1	MMD	64	F	Cilostazol 100 mg/day, atorvastatin 10 mg/day	Cefazolin 1 g	ICH	2 months	One side	NR	NR
2	MMD	47	F	Aspirin 100 mg/day, Lansoprazole 15 mg/day, Atorvastatin Calcium Hydrate 10 mg/day, Tsumura Goreisan 2.5g/day, Bepotastine Besilate 10 mg/day	Cefazolin 1 g	IF	8 months	Bilateral	NR	NR
3	MMD	48	F	Amlodipine Besilate 5 mg/day	Cefazolin 1 g	ICH	6 months	Bilateral	NR	NR
4	MMD	49	F	Aspirin 100 mg/day	Cefazolin 1 g	TIA	9 months	Bilateral	NR	NR
5	MMD	59	F	Cilostazol 100 mg/day, Esomeprazole Magnesium Hydrate 20 mg/day	Cefazolin 1 g	IF	11 months	Bilateral	NR	NR
6	MMD	50	F	Cilostazol 100 mg/day, Olopatadine Hydrochloride 5 mg/day	Cefazolin 1 g	TIA	1 month	Bilateral	NR	NR

7	MMD	45	F	Aspirin 100 mg/day, Lansoprazole 15 mg/day, Clopidogrel Sulfate 75 mg/day, Pitavastatin Calcium Hydrate 2 mg/day	Cefazolin 1 g	IF	1 year and 1 month	Bilateral	NR	NR
8	MMD	48	F	Aspirin 100 mg/day, Lansoprazole 15 mg/day, Celecoxib 100 mg/day	Cefazolin 1g	TIA	3 months	Bilateral	NR	NR
9	MMD	51	M	Aspirin 100 mg, Etizolam 0.5 mg/day, Olmesartan Medoxomil 10 mg/day, Azelnidipine 8 mg/day	Cefazolin 1 g	TIA	1 month	Bilateral	NR	NR
10	MMD	53	F	Aspirin 100 mg/day, Lansoprazole 15 mg/day	Cefazolin 1 g	TIA	7 months	Bilateral	NR	NR
11	MMD	43	F	Aspirin 100 mg, /day Lansoprazole 15 mg/day, Telmisartan 40 mg/day	Cefazolin 1 g	TIA	1 year and 2 months	Bilateral	NR	NR
12	MMD	34	F	Aspirin 100 mg/day	Cefazolin 1 g	TIA	5 months	Bilateral	NR	NR
13	MMD	29	F	Levetiracetam 1000 mg/day, Phenytoin 300 mg/day, Sodium Ferrous Citrate 100 mg/day	Cefazolin 1 g	ICH	7 months	Bilateral	NR	NR
14	MMD	32	M	Aspirin 100 mg/day, Lansoprazole 15 mg/day	Cefazolin 1 g	TIA	Several days	Bilateral	NR	NR
15	MMD	7	F	NR	Cefazolin 1 g	TIA	Several days	Bilateral	NR	NR

16	MMD	27	M	Aspirin 100 mg/day, Esomeprazole Magnesium Hydrate 20 mg/day	Cefazolin 1 g	TIA	8 months	Bilateral	NR	NR
17	MMD	5	M	Aspirin 30 mg/day	Cefazolin 0.3 g	IF	3 months	Bilateral	NR	NR
18	MMD	39	M	NR	Cefazolin 1 g	TIA	7 months	Bilateral	NR	NR
19	MMD	11	M	Cilostazol 150 mg/day	Cefazolin 0.5 g	TIA	2 months	Bilateral	NR	NR
20	MMD	46	F	Sodium Ferrous Citrate 50 mg/day, Olopatadine Hydrochloride 10 mg/day	Cefazolin 1 g	TIA	2 months	Bilateral	NR	NR
21	MMD	35	F	Pregabalin 225 mg/day, Amitriptyline Hydrochloride 35 mg/day, Desloratadine 5 mg/day	Cefazolin 1 g	TIA	8 months	Bilateral	NR	NR

22	IA	65	F	Clopidogrel Sulfate 75 mg/day, Atorvastatin Calcium Hydrate 10 mg/day, L-Carbocisteine 1500 mg, Mecobalamin 1500 μg/day, d-Chlorpheniramine Maleate 6 mg/day, Betamethasone 0.5mg/day, d-Chlorpheniramine Maleate 4 mg/day, Olopatadine Hydrochloride 20 mg/day	Cefazolin 1 g	Oculomotor nerve palsy, head ache	Several days	NR	Cavernous/27mm	NR
23	IA	71	F	Aspirin 100 mg/day, Esomeprazole Magnesium Hydrate 20 mg/day, Valsartan 80 mg/day, Amlodipine Besilate 5 mg/day, Suvorexant 15 mg/day	Cefazolin 1 g	NR	NR	NR	ICA/21mm	NR
24	IA	62	F	Loxoprofen Sodium Hydrate 180 mg/day, Rebamipide 300 mg/day, Brotizolam 0.25 mg/day, Diazepam 2 mg/day	Cefazolin 1 g	None	NR	NR	ICA/27mm	NR
25	IA	78	F	Etizolam 0.5 mg/day, Tandospirone Citrate 20 mg/day, Kallidinogenase 100 units/day, Magnesium Oxide 660 mg/day	Cefazolin 1 g	None	NR	NR	Cavernous/28mm	NR

26	IA	79	F	Aspirin 100 mg/day, Cilostazol 100 mg/day, Acetaminophen 1200 mg/day, Mecobalamin 1500 μg/day, Benidipine Hydrochloride 4 mg/day, Ezetimibe 10 mg/day, Rosuvastatin Calcium 5 mg/day, Candesartan Cilexetil 8 mg/day	Cefazolin 1 g	ONP	1 month	NR	Cavernous/26mm	NR
27	IA	70	F	Valsartan 160 mg/day, Amlodipine Besilate 10 mg/day, Imidapril Hydrochloride 5 mg/day, Doxazosin Mesilate 2 mg/day, methotrexate 10 mg/week	Cefazolin 1 g	ONP	Several days	NR	Cavernous/20mm	NR
28	EPI	56	M	Levetiracetam 2000 mg/day, Lamotrigine 300 mg/day, Perampanel Hydrate 4 mg/day, Pravastatin Sodium 20 mg/day	Cefazolin 1 g	Seizures	Several days	NR	NR	Temporal lobe
29	EPI	14	M	Lacosamide 300 mg/day, Clobazam 15 mg/day, Perampanel Hydrate 6 mg/day	Cefazolin 1 g	Seizures	Several days	NR	NR	Frontal lobe
30	EPI	20	M	Lacosamide 400 mg/day, Lamotrigine 200 mg/day,	Cefazolin 1 g	Seizures	Several days	NR	NR	Frontal lobe

				Carbamazepine 400 mg/day						
31	EPI	43	F	Carbamazepine 300 mg/day, Atorvastatin Calcium Hydrate 5 mg/day, Fexofenadine Hydrochloride 120 mg/day	Cefazolin 1 g	Seizures	Several days	NR	NR	Temporal lobe
32	EPI	2	M	Levetiracetam 325 mg/day, Zonisamid 16 mg/day	Cefotaxime 0.5 g	Seizures	Several days	NR	NR	Frontal lobe
33	MMD	64	F	Atorvastatin 10 mg/day	Cefazolin 1 g	ICH	2 months	Bilateral	NR	NR
34	MMD	47	F	Aspirin 100 mg/day, atorvastatin 10 mg/day	Ceftriaxone 2 g	TIA	5 months	Bilateral	NR	NR
35	MMD	48	F	Amlodipine 5 mg/day	Ceftriaxone 2 g	ICH	6 months	Bilateral	NR	NR
36	MMD	49	F	Aspirin 100 mg/day	Cefazolin 1 g	TIA	6 months	Bilateral	NR	NR
37	MMD	59	F	Cilostazol 200 mg/day	Cefazolin 1 g	IF	11 months	Bilateral	NR	NR
38	MMD	50	F	Cilostazol 200 mg/day	Cefazolin 1 g	IF	8 months	Bilateral	NR	NR
39	MMD	45	F	Aspirin 100 mg/day, clopidogrel 75 mg/day	Clindamycin 600 mg	TIA	1 month	Bilateral	NR	NR
40	MMD	48	F	Aspirin 100 mg/day	Cefazolin 1 g	TIA	4 months	Bilateral	NR	NR

41	MMD	51	M	Aspirin 100 mg/day, olmesartan 20 mg/day, azelnidipine 16 mg/day, pitavastatin 2 mg/day	Cefazolin 1 g	TIA	2 months	Bilateral	NR	NR
42	MMD	53	F	Aspirin 100 mg/day	Cefazolin 1 g	TIA	4 months	Bilateral	NR	NR
43	MMD	43	F	Aspirin 100 mg/day, telmisartan 80 mg/day	Cefazolin 1 g	TIA	10 months	Bilateral	NR	NR
44	IA	62	F	NR	Cefazolin 1 g	NR	NR	NR	Supraclinoid/ 27 mm	NR
45	IA	78	F	NR	Cefazolin 1 g	Oculomotor nerve palsy	5 months	NR	Cavernous/ 30 mm	NR
46	IA	79	F	Cilostazol 100 mg/day, candesartan 8 mg/day, benidipine 4 mg/day, rosuvastatine 5 mg/day, ezetimibe 10 mg/day	Cefmetazole 1 g	Oculomotor nerve palsy	6 months	NR	Cavernous/ 25 mm	NR
47	IA	70	F	Clopidogrel 75 mg/day, valsartan 160 mg/day, amlodipine 10 mg/day, imidapril 5 mg/day, doxazosin 2 mg/day, methotrexate 10 mg/week	Cefazolin 1 g	Oculomotor nerve palsy	7 months	NR	Cavernous/ 20 mm	NR
48	IA	65	F	Clopidogrel 75 mg/day, atorvastatin 10 mg/day	Cefazolin 1 g	NR	NR	NR	Cavernous/ 26 mm	NR
49	IA	71	F	Aspirin 100 mg/day, valsartan 160 mg/day, amlodipine 5 mg/day	Cefazolin 1 g	NR	NR	NR	Supraclinoid/ 22 mm	NR
50	EPI	56	M	Pravastatin 20 mg, levetiracetam 2000 mg/day, lamotrigine 300 mg/day, perampanel 4 mg/day	Cefazolin 1 g	Seizures	Several days	NR	NR	Temporal lobe

51	EPI	14	M	Lacosamide 300 mg/day, perampanel 6 mg/day, clobazam 15 mg/day	Cefazolin 1 g	Seizures	Several days	NR	NR	Frontal lobe
52	EPI	20	M	Lacosamide 300 mg/day, lamotrigine 200 mg/day, carbamazepine 400 mg/day	Cefazolin 1 g	Seizures	Several days	NR	NR	Frontal lobe

Abbreviations: MMD, moyamoya disease; F, female; M, male; IA, intracranial aneurysm; EPI, epilepsy; ONP, ocular nerve palsy; ICH, intracerebral hemorrhage; IF, infarction; TIA, transient ischemic attack; ICA, internal carotid artery; MCA, middle cerebral artery; NR, not reported.

Table S2：Detailed clinical information for the participants of discover cohort.

ID	Age	Sex	Disease	Hypertension	Diabetes	Coronary heart disease	Smoke	Alcohol	Clinical presentation	Duration (mounth)	Suzuki stge	Subtype of MMD
1	45	M	MMD	N	N	N	N	N	ICH	9	3	Bilateral
2	32	M	MMD	Y	Y	N	N	Y	ICH	5	4	Bilateral
3	47	M	MMD	Y	Y	N	Y	Y	TIA	8	4	Bilateral
4	46	M	MMD	Y	N	N	N	N	TIA	3	3	Bilateral
5	51	F	MMD	Y	N	N	N	N	TIA	36	3	Bilateral
6	35	F	MMD	N	N	N	N	N	ICH	5	4	Bilateral
7	36	F	MMD	N	N	N	Y	N	TIA	0.5	4	Bilateral
8	52	M	MMD	Y	N	N	N	Y	TIA	3	4	Bilateral
9	52	M	MMD	N	N	N	N	N	TIA	6	3	Bilateral
10	31	F	MMD	N	N	N	N	N	TIA	4	3	Bilateral
11	52	M	IA	Y	N	N	N	N	ICH	N	N	N
12	51	M	IA	N	N	N	Y	Y	ICH	N	N	N
13	49	F	IA	N	N	N	N	N	ICH	N	N	N

Abbreviations: MMD, moyamoya disease; F, female; M, male; IA, intracranial aneurysm; ICH, intracerebral hemorrhage; TIA, transient ischemic attack; N, not reported； Y， be reported.

Table S3: The list of 198 differentially expressed genes.

Gene	logFC	AveExpr	T	P.Value	B
KCTD10	-2.434440911	3.610096775	-6.731081169	5.66E-09	10.20141432
HLA-A	2.122370703	6.320964854	6.528574162	1.27E-08	9.455333609
FADS3	-2.836148994	4.287760661	-6.063982593	8.05E-08	7.757182976
UBE2W	-1.68296515	1.625124795	-5.676184373	3.66E-07	6.360646239
C5orf24	-1.61269155	1.826951474	-5.654164023	3.99E-07	6.282077009
ZDBF2	-1.573127072	1.066455813	-5.402476422	1.05E-06	5.390613772
CLIC4	-1.653373145	5.462060266	-5.302016453	1.54E-06	5.038463269
BRIX1	-2.18600279	2.845543638	-5.271296195	1.73E-06	4.931231468
PI16	-3.190028005	2.986982328	-5.225874162	2.05E-06	4.77308641
COL15A1	-1.878930102	1.166109273	-5.211171244	2.17E-06	4.722000889
ITPRIPL2	-1.893584842	6.109663305	-5.167770166	2.55E-06	4.571510071
ODC1	-1.945920377	7.439824676	-5.117291599	3.09E-06	4.397067847
REEP1	-1.776215059	1.431697911	-5.06133925	3.81E-06	4.20447226
LGALS9C	2.989663687	2.371814005	5.043977177	4.06E-06	4.144876626
GCOM1	-1.552695988	2.107342814	-4.991019091	4.95E-06	3.963597411
TMSB10	2.116562132	9.099582895	4.946769621	5.83E-06	3.812718908
PPP2R2B	-1.99196893	3.821920642	-4.882060303	7.41E-06	3.593075227
ARIH1	-1.503263429	2.35364489	-4.878812556	7.49E-06	3.582083214
MSRB3	-2.037593118	6.71240435	-4.853091045	8.24E-06	3.495138049
RHOQ	-1.773606341	3.427806232	-4.849395883	8.35E-06	3.482663524
DSTNP2	-1.512623709	6.720729201	-4.815892653	9.44E-06	3.369745775
RARRES3	2.281476076	5.882619418	4.807039518	9.76E-06	3.339964017
PJA2	-1.664413537	5.695605016	-4.802270057	9.93E-06	3.323929508
HIF1AN	-1.76275187	3.01997113	-4.782277388	1.07E-05	3.256791496
RAI2	-1.92682003	2.128359834	-4.761972952	1.15E-05	3.188732406
AMN	1.703872353	3.339175943	4.754752235	1.18E-05	3.164559882
S100A10	2.484899967	8.054994918	4.745434995	1.22E-05	3.133392958
LPIN3	1.914942216	2.292166034	4.691101435	1.49E-05	2.952189206
STK24	-1.51984884	1.679391394	-4.668002135	1.62E-05	2.875439254
GNPDA2	-1.569157768	1.162450388	-4.638087123	1.80E-05	2.776302231
PTP4A1	-1.885999203	4.708631951	-4.608447582	2.01E-05	2.67837022
FAM122A	-1.709503442	2.451089949	-4.605596153	2.03E-05	2.668964301
PTPN11	-1.575573812	4.052954906	-4.60471308	2.03E-05	2.666051893
ARNT	-1.666704461	2.119629415	-4.597947548	2.08E-05	2.643747609
SPECC1L	-1.768875935	4.150386207	-4.589648471	2.15E-05	2.616408715
HSPA4	-1.539954411	2.403148982	-4.530575713	2.65E-05	2.422490763
KCNAB1	-1.770660704	1.869074105	-4.525741989	2.70E-05	2.406676514
RPS29	1.605593911	6.309009592	4.502959656	2.93E-05	2.332250981
SLC20A1	1.864387916	5.594171616	4.502524638	2.93E-05	2.330831638
USP22	-1.838931935	2.598452989	-4.45246772	3.50E-05	2.167959987
CISD3	1.932935113	4.829360102	4.405835059	4.13E-05	2.017045244

UBQLN2	-1.636551979	3.138137123	-4.39857133	4.24E-05	1.993609969
NUDT8	1.790323614	3.551069458	4.388663253	4.39E-05	1.961674691
NEO1	-2.173068395	2.157777671	-4.377734839	4.56E-05	1.926493141
HLA-J	1.507908185	7.956930543	4.366856975	4.74E-05	1.891518743
NEU1	1.707322246	2.249504127	4.365659437	4.76E-05	1.887671147
TWSG1	-1.688151808	5.851416982	-4.352296607	4.99E-05	1.844774132
TBC1D9B	1.96852414	2.394867113	4.352095421	4.99E-05	1.844128807
OR51E2	-1.661531837	2.47932977	-4.339659918	5.22E-05	1.804270383
DUSP3	-1.703700855	2.665219879	-4.32999868	5.40E-05	1.773344718
AQP5	1.614051648	1.5781087	4.263057779	6.82E-05	1.560056141
ROCK2	-1.704889659	4.909850957	-4.261617342	6.85E-05	1.555485817
PURA	-2.216117228	5.358359943	-4.258956562	6.91E-05	1.547045651
ZNHIT1	1.587089982	5.078924767	4.251434565	7.10E-05	1.523200529
IPO7	-1.685229503	5.127026206	-4.230244886	7.64E-05	1.456148911
MYOM1	-2.015694458	4.715464239	-4.22088382	7.89E-05	1.426584281
HLA-B	1.654166239	7.703246743	4.21902179	7.94E-05	1.420707707
VPS13A	-1.756302903	2.882970198	-4.21416877	8.08E-05	1.405398106
MAP2K1	-2.114996561	3.417020386	-4.199088433	8.51E-05	1.35788547
HTATSF1	-1.837833283	5.108283886	-4.185024932	8.93E-05	1.313659564
AMIGO2	-2.096331461	4.573327731	-4.172958177	9.31E-05	1.275777155
UCHL1	-2.263026323	4.01802249	-4.171563861	9.35E-05	1.271403674
MDK	2.870164715	5.757759855	4.146258227	0.000102043	1.192167814
CASQ2	-1.601236806	3.201284816	-4.131093844	0.000107488	1.14481281
DDX6	-1.943014528	4.335684024	-4.122962153	0.000110523	1.119458826
NEXN	-2.138084945	6.78150102	-4.097506107	0.000120567	1.040268249
FZD1	-2.155314154	4.608321489	-4.077574486	0.00012904	0.978454723
ISG15	1.644773976	2.496335422	4.074219727	0.000130522	0.968067307
F7	1.877358612	4.3423798	4.069417174	0.000132671	0.953205423
TMEM238	1.606678648	4.833235559	4.06653833	0.000133976	0.944301344
SPIN1	-1.592605885	1.942039348	-4.047529905	0.000142907	0.885598865
ISG20	2.774502997	3.854680241	4.044192329	0.000144533	0.87530772
ENPP2	-1.669446265	1.540128661	-3.998117448	0.00016889	0.733734906
MXRA8	-1.770853294	2.488438165	-3.988678356	0.000174347	0.704846627
TEX2	-1.575832588	2.541515534	-3.98636985	0.000175707	0.697787454
UBL3	-1.528732184	5.196210217	-3.971285628	0.000184848	0.65171966
LBH	-2.521037478	3.412215557	-3.941239089	0.000204438	0.56025909
HLA-C	1.946401821	8.711874532	3.935724324	0.000208244	0.543516422
NUDT3	-1.732999624	4.256952978	-3.875910197	0.000254152	0.362811866
STAG3L2	-2.017506392	5.169085577	-3.865935349	0.000262697	0.332836856
LINC00111	1.864386442	2.148699995	3.864129969	0.000264273	0.327416515
EGR2	2.757492056	7.576362577	3.850602427	0.000276373	0.286850704
AP3M1	-1.519036032	1.587062202	-3.835953561	0.000290074	0.243018768
HSPB7	-1.909420362	2.37569556	-3.835257867	0.00029074	0.240939634
CSRP1	-1.606077855	4.222801628	-3.802292686	0.000324067	0.142682653

PATZ1	1.923416047	2.146924797	3.792728403	0.000334402	0.114271603
IQGAP2	1.709657953	4.996863681	3.790419975	0.000336944	0.107420874
STXBP1	1.724551004	4.041133243	3.786138177	0.000341706	0.094720521
UXT	1.51894996	3.771313545	3.779214647	0.000349544	0.07420304
CASP4	2.101062451	3.835738041	3.769639535	0.000360668	0.045865698
PREPL	-1.556269144	4.556521086	-3.755973173	0.000377134	0.005496973
CDC42EP5	1.733856368	7.662027457	3.752448951	0.000381495	-0.004898484
ICAM3	2.43750898	3.427053997	3.749708566	0.000384919	-0.012977676
SUSD5	-2.063066486	5.263420651	-3.734233792	0.000404816	-0.058531809
POLD4	1.623714583	6.059170519	3.706182643	0.000443416	-0.140809217
PJA1	-1.683330597	4.138582034	-3.688571962	0.00046942	-0.192265285
SERPINE1	-2.223980241	1.819234981	-3.683935606	0.000476504	-0.205786523
ALDH1B1	-2.527535175	4.688509745	-3.680431249	0.000481926	-0.215999356
C11orf91	1.66799267	1.764293241	3.663052947	0.000509698	-0.26655481
PPP1R3C	-1.635362465	9.490844811	-3.660093843	0.000514576	-0.275148117
MYO1F	1.833841641	1.838500567	3.649891547	0.000531739	-0.304742095
TMEM47	-1.717424691	2.41715926	-3.648999061	0.000533266	-0.307328452
SNORD99	1.865809238	1.631067498	3.646456295	0.00053764	-0.314694997
FRZB	-2.039021115	6.988497696	-3.640605184	0.000547835	-0.331633619
SPOCK1	-1.80534003	1.740234857	-3.638881884	0.000550873	-0.336619175
GAR1	-1.894651498	5.103896929	-3.620748116	0.000583826	-0.388989409
PSMB9	1.610990971	4.753865229	3.619891357	0.000585428	-0.391459591
C19orf24	2.06337692	5.313599308	3.618003944	0.000588974	-0.396900007
KCNA5	-2.410438852	8.154958497	-3.615209419	0.00059426	-0.404951809
GJA1	-2.12961406	2.357568764	-3.613730626	0.000597076	-0.409211011
PTBP2	-1.925186637	4.899003697	-3.606122064	0.000611765	-0.431107442
ID1	1.701594001	5.060458812	3.593085541	0.000637737	-0.468555891
HEY2	-1.907173231	4.026013348	-3.587593457	0.000648989	-0.484306197
RPL32	1.514881899	7.007179004	3.583890864	0.000656682	-0.494915796
NOV	-2.678802216	8.956365312	-3.575889426	0.000673604	-0.517819352
S100A4	1.724148618	13.67862157	3.54992858	0.00073141	-0.591901865
ADPRH	-1.53806275	2.817372538	-3.529989916	0.000778976	-0.648560544
PTRHD1	1.964285097	3.232957394	3.510643096	0.00082793	-0.703337573
INSR	1.672577729	5.382177987	3.498456697	0.000860251	-0.73773952
TANC1	-1.727760083	4.71444017	-3.490447186	0.000882142	-0.76030722
DKC1	-1.770275889	3.671320699	-3.488005255	0.00088892	-0.767180834
SRL	-1.731900308	2.668944715	-3.462965872	0.000961338	-0.837478316
LPP	-1.876715626	4.839779199	-3.457872062	0.000976739	-0.851737853
MGAT4B	2.084790491	3.747410409	3.451649519	0.00099587	-0.869138165
TBL2	-1.676693083	2.962685723	-3.443316214	0.001022048	-0.892408094
H2AFX	2.091308226	3.84788284	3.436233329	0.001044809	-0.912156768
KCNJ2	2.050080043	2.723670884	3.427729141	0.001072771	-0.935832348
CD55	2.223940805	6.873876133	3.402593258	0.00115962	-1.005579665
PITPNB	-1.545053706	5.73619803	-3.401717561	0.001162762	-1.008003316

AKAP12	-1.517489658	2.728789329	-3.376847215	0.001255446	-1.076660176
TGM2	-2.164991137	5.400765899	-3.376520037	0.001256711	-1.077561102
RAB36	1.666624651	4.27225516	3.354526117	0.001344535	-1.13798782
IL1RN	1.659869179	1.68021866	3.35182165	0.001355726	-1.145399542
VARS2	1.964206014	2.053760261	3.350216623	0.001362409	-1.149796264
CFL2	-1.719212065	5.940522448	-3.337541849	0.001416288	-1.184466182
ARAP3	2.018555097	3.102163881	3.334551376	0.00142929	-1.192633029
DBN1	-1.817294165	5.647589452	-3.318922145	0.001499096	-1.235233875
BASP1	-2.047545484	5.884128606	-3.318405474	0.001501458	-1.236639821
ENDOD1	-1.58474671	2.944788094	-3.307463707	0.001552303	-1.266378697
CDA	1.838281699	2.101975401	3.287536855	0.001649095	-1.320363789
FLNC	-1.877827896	4.734834878	-3.286028179	0.00165665	-1.324441827
JMJD7-PLA2G4B	1.925562816	3.601368917	3.268740957	0.00174558	-1.371077286
C5orf22	-1.521502366	1.615207104	-3.265451045	0.001763005	-1.379933024
EVI2B	1.513664013	1.294477184	3.249113715	0.001851987	-1.423817329
NMUR1	1.719864784	3.80629872	3.238373751	0.001912765	-1.45258238
ITGB4	2.101842937	2.204526791	3.233024293	0.001943733	-1.466884999
DNTTIP2	-1.618382511	4.964248044	-3.230970003	0.00195575	-1.47237305
IRF7	1.896179876	3.565638593	3.225802555	0.001986287	-1.486167086
C4orf48	1.934680612	4.213090021	3.224084914	0.001996537	-1.490748731
RASSF3	-1.553123633	6.784690668	-3.223536108	0.001999822	-1.492212259
ERG	-1.67429053	4.349707902	-3.207233394	0.00209976	-1.535607309
PMM1	1.528828993	2.628034858	3.20188323	0.002133566	-1.549814657
ADAM8	1.832568869	1.635873784	3.201382132	0.002136759	-1.551144462
SERTAD1	1.907324687	6.987015887	3.172933499	0.002325527	-1.626398685
FRAT2	2.363797945	2.476718653	3.163397366	0.002392225	-1.651517313
LATS2	-1.624439205	5.606548852	-3.152200761	0.002472825	-1.680940788
PPP2CB	-1.505428075	5.760161108	-3.15118508	0.002480261	-1.683606198
ADM	1.777327352	8.703310263	3.146618555	0.002513952	-1.695582345
ZFAT-AS1	1.691794302	1.58821196	3.130808636	0.002633936	-1.736949134
CD24	1.500985622	2.189683211	3.119018764	0.002726876	-1.767699908
SYNM	-1.52891185	7.899047681	-3.100115707	0.002882314	-1.816828842
SNORA57	1.572942407	2.194568308	3.085063534	0.003011966	-1.855794719
C16orf45	-1.666628359	4.406514313	-3.082365323	0.003035775	-1.86276509
SNORD114-11	1.661726467	2.439565878	3.079554192	0.003060767	-1.870022458
MICALL2	1.508952005	2.526601718	3.075685268	0.003095478	-1.880002812
DCN	-1.691532209	8.11052299	-3.059917637	0.003240774	-1.920582781
MAP3K11	1.653703553	2.863477264	3.049586052	0.003339401	-1.947089841
TTLL7	-1.533867368	3.154942148	-3.044508407	0.003388892	-1.960093187
KAT2A	1.807934477	3.098067307	3.025479844	0.003580504	-2.008682064
SMC3	-2.022014731	4.853452691	-3.019127165	0.003646688	-2.024853597
TSPAN8	-1.813952494	4.367490628	-2.99177864	0.003944824	-2.094186246
VSIG2	2.106327688	3.803489967	2.975668038	0.004130899	-2.134810409

TGFB3	-1.656627566	5.458116129	-2.969091883	0.00420917	-2.151345888
RBPMS2	-1.735136782	4.161719274	-2.96193408	0.004295928	-2.169312958
NES	1.530901522	3.237544489	2.959153025	0.004330082	-2.176285076
TNFRSF10D	1.763382158	4.473768723	2.947770444	0.004472513	-2.204770333
IRF1	1.806648846	2.721790429	2.940303821	0.00456829	-2.223411233
APOL1	1.526368575	6.154785656	2.937230248	0.004608266	-2.231074329
UBE2E1	-1.684193832	2.423535026	-2.927238784	0.004740469	-2.25594376
CKMT2	-1.598381841	2.478377255	-2.926377522	0.004752028	-2.258084525
SRSF8	-1.830267431	6.837068062	-2.920584623	0.004830454	-2.272471145
IFI27L2	1.532479724	5.114376564	2.888334894	0.005289412	-2.352170564
EIF4EBP1	1.60850136	4.066616443	2.863572809	0.005668819	-2.412911223
GPR4	1.548075718	1.919108307	2.858575096	0.00574838	-2.425122313
IFITM1	1.521705933	6.456338737	2.83127646	0.006201443	-2.491535079
TCIRG1	1.710787068	7.582108836	2.822155725	0.006360004	-2.513615686
MYOZ1	-1.655670711	3.191145569	-2.806552929	0.006639939	-2.551262221
SLC9A3R1	2.179668774	4.894500587	2.799113135	0.006777372	-2.569156558
C1S	-1.829602041	3.205845675	-2.784725648	0.007050599	-2.603657859
SDC4	-2.069533802	6.682646603	-2.768094311	0.007379035	-2.643368873
UFSP2	-1.509762968	5.909245854	-2.766047593	0.00742041	-2.648243156
ITGA10	1.523090604	4.019640615	2.747782966	0.007799178	-2.69161677
CCND1	-1.722460014	5.73879702	-2.747644487	0.007802117	-2.69194477
PODXL	1.65237118	6.801249243	2.740932951	0.007945747	-2.707826182
BCL3	1.535349455	6.159366511	2.721965716	0.008364859	-2.752544519
DNAJA4	-1.619101669	2.832810778	-2.67565409	0.009474587	-2.860709926
HBA2	-3.266061622	6.500286756	-2.662841571	0.009804506	-2.890377041
GNG13	1.789736587	2.395332606	2.659374237	0.009895573	-2.898386262
